# Identifying neural correlates of multidimensional, subjective gaming experiences during active gameplay

**DOI:** 10.3389/fnhum.2022.1013991

**Published:** 2022-10-28

**Authors:** Uijong Ju

**Affiliations:** HXR Lab, Department of Information Display, Kyung Hee University, Seoul, South Korea

**Keywords:** gaming experience, fMRI, correlational analysis, competence, tension

## Abstract

Studying how gaming experiences are encoded is important to understand the effects of gaming on the brain. Although studies have investigated neural correlates of gaming experiences, the brain patterns related to the full range of subjective experiences across different types of games are yet to be identified. The present study used three custom-made, immersive driving games with different input dynamics (controlling a car, a boat, or a spaceship) and different mechanics to assess subjective gaming experiences in a magnetic resonance imaging scanner. A correlational analysis identified several brain networks associated with different subjective gaming experiences, including visual and attentional processing networks. The contributions of these networks were further validated using meta-analysis-based functional term decoding. The results of the present study point to a range of perceptual, motivational, and control networks that are engaged during active gameplay.

## Introduction

The computer game industry is one of the fastest growing industries of the 21st century. Its explosive development over the past two decades has been accompanied by profound changes in how people enjoy computer and video games. Although earlier, most people played a game for a specific reward or a specific goal, nowadays the focus is on casual gaming that is regarded as just one leisure activity among others (Kuittinen et al., 2007), as well as on games that are interesting and keep the player motivated (Przybylski et al., 2009, 2010). With these changes in the focus and developments in the gaming industry, more people enjoy games, and gaming experiences have become a common leisure experience in young adults (Padilla-Walker et al., 2010). However, limited neuroimaging studies have investigated the relationship between gaming experiences and neural activity that can potentially be the link to understand both the good and bad effects of gaming experiences, like the mental treatment of attention deficit hyperactivity disorder (ADHD) through video games which is approved by United States Food and Drug Administration (FDA) (Canady, 2020), and “game addiction” that has officially been added as a mental health disorder by the World Health Organization (WHO) (Pontes et al., 2021).

In order to study the relationship between neural activity and gaming experiences, first, it is important to reflect upon a complete picture of gaming experiences in experiments in order to investigate which brain regions are related to the players’ subjective experiences. The Gaming Experience Questionnaire (GEQ) (IJsselsteijn et al., 2013) is one of the representative questionnaires used to measure gaming experiences in seven different dimensions that captures a wide range of aspects, including competence, immersion, flow, tension, challenge, and positive and negative effect. Based on a meta-analysis, they have a reliability between 0.69 and 0.91 (mean = 0.81) and validity between 0.38 and 0.66 (mean = 0.49) (Law et al., 2018). Since the present study uses the GEQ, each dimension has been briefly defined and summarized in the following text, in the context of potential brain regions associated with each dimension.

*Competence* serves as a criterion for evaluating a player’s subjective evaluation of their own performance in the game (White, 1959). This is crucial for the gaming experience, since “excellent games” tend to increase players’ competence while also keeping them engaged without causing aggravation (Gee, 2003). Previous research has shown by measuring electroencephalography (EEG), when playing virtual reality (VR) games that frontal alpha activity negatively correlated with competence (Benlamine et al., 2021) and general competence traits were associated with the ventromedial prefrontal cortex and the precuneus (Ma et al., 2016). Additionally, competence related feature of positive feedback about performance showed increased activation of the lingual gyrus in contrast to receiving negative feedback (Drueke et al., 2015).

*Immersion* refers to a player becoming more involved and absorbed in a game to the point where they may even lose track of their surroundings (Jennett et al., 2008). Previous research has shown that the insula and insula-related self-awareness and sensory attention process regions have been linked to game immersion (Ju and Wallraven, 2019). Additionally, immersion related experiences of a sense of presence (Nacke and Lindley, 2008) in VR studies found that prefrontal regions (Baumgartner et al., 2006), and activity of left and right dorsolateral prefrontal cortex (DLPFC) were associated with upregulating and downregulating of sense of presence (Baumgartner et al., 2008; Clemente et al., 2013).

*Flow* is defined as a state of concentration in which people are totally engrossed in an activity (Csikszentmihalyi, 1990). The difference between immersion and flow is that immersion is a progressive experience, and that people who are immersed may still be aware of their surroundings (Nacke and Lindley, 2008). Flow-related regions have been investigated in video game studies that found that frontal networks (Yoshida et al., 2014; de Sampaio Barros et al., 2018), reward, sensorimotor networks (Klasen et al., 2011), and visual and attentional processing related networks (Ju and Wallraven, 2019) were associated with flow.

*Tension* is defined as a state of pressure and anxiety that makes it difficult to relax. Tension has positive aspects, in that a feeling of high tension can be related to a flow state in which players feel they are part of a gaming situation; this can reduce tension or anxiety caused by outside factors (Garakani et al., 2021; Pallavicini et al., 2021). In contrast to other experiences, tension during gaming has not been investigated alone, whereas in comparison to the flow states, when participants were in anxiety state, brain activity in the frontal-temporal region decreased (Yelamanchili, 2018). General tension induced by music has been shown to be associated with the left lateral orbitofrontal cortex as well as the amygdala (Lehne et al., 2013).

*Challenge* is usually described as a new or difficult task that tests a person’s expertise and ability. It is crucial to match a game’s difficulty level to the player’s ability (Spronck et al., 2004), since challenges can enhance motivation and learning during educational games (Burnes et al., 2015; Hamari et al., 2016). Challenge-related brain regions have been related to reward networks in the midbrain structures; additionally, the cerebellum, thalamus, parietal and occipital areas as well as the premotor cortex have shown activation during high-challenge states while the cuneus showed activation during low-challenge states (Klasen et al., 2011). Additionally, higher-level visual areas and the fusiform gyrus have been shown to be associated with challenge (Ju and Wallraven, 2019).

*Positive and negative affect* are functional terms related to a person’s well-being (Andrews and Withey, 2012). Positive affect is represented by feelings of joy, engagement, and alertness, whereas negative affect is associated with unpleasant feelings of distress (Watson, 1988). Positive and negative affect has been found to correlate with the sympathetic and parasympathetic systems (Bruya, 2010) whereas in gaming experiences, negative affect has been shown to be positively correlated with bilateral ventromedial prefrontal cortex (Mathiak et al., 2013) and negatively correlated with dorsal and ventral visual streams (Ju and Wallraven, 2019) and visual-related areas including precuneus (Mathiak et al., 2013; Ju and Wallraven, 2019). In contrast, positive affect ratings are positively correlated with reward-related networks (Kätsyri et al., 2013) and negatively correlated with activity of bilateral insula (Mathiak et al., 2013).

Already several studies have tried to find brain regions associated with subjective gaming experiences, however, investigations of subjective gaming experiences were usually limited to one or two dimensions and resulting brain regions for same gaming experience were inconsistent across studies. For instance, previous studies have investigated brain regions associated with a single gaming experience such as flow (Baumgartner et al., 2006, 2008; Clemente et al., 2013), and found that main brain regions for decoding flow experiences were varied across studies, including the somatosensory and motor regions (Klasen et al., 2011), prefrontal regions (Yoshida et al., 2014; de Sampaio Barros et al., 2018) and visual processing related regions (Ju and Wallraven, 2019) being the main regions associated with flow. Additionally, even though some gaming experience studies used two contrast dimensions like positive and negative affect and investigated brain regions to decode such experiences, studies found that regions for contrast experiences were different. Reward systems (Kätsyri et al., 2013), and insula (Mathiak et al., 2013) were correlated with positive affect whereas frontal, visual, dorsal regions were associated with negative affect (Mathiak et al., 2013) which implied that different brain regions contribute to encode contrasting experiences. Overall, variability of gaming experiences in previous studies implied that simple investigation of one or two experiences was not enough to understand complex gaming experiences.

Going beyond this research focusing on limited gaming experiences, in a previous study we aimed to investigate the full range of gaming experiences (based on the seven dimensions of the GEQ) using game context manipulation (Ju and Wallraven, 2019). In this previous study, we used a car game and manipulated the number of targets, obstacles, and acceleration to find brain regions associated with differences in subjective gaming experiences between conditions. We found that visual, attentional, and sensorimotor networks contributed to the decoding of immersion, flow, challenge, and negative affect. However, since we limited the games to car games, it is difficult to generalize our findings to other games with different control inputs. Additionally, we were only able to identify four of the seven gaming experiences, which indicates that our in-context manipulations were not far-reaching enough to encode all subjective gaming experiences. In order to solve this problem, the present study used three different types of vehicles to find common brain networks associated with different types of vehicle games. Additionally, given the complexity of subjective experiences and the resulting activation patterns we identified in our previous study (Ju and Wallraven, 2019), the number of different brain areas related to attentional, visual, sensorimotor, and emotional processing potentially involved in the gaming experiences in this study were expected to make the analyses potentially difficult. This study therefore used several different techniques to address this issue, including correlational analyses, a meta-analysis tool [Neurosynth (Yarkoni et al., 2011)] to identify functional correlates as well as recent meta-analysis tool [NeuroQuery (Dockès et al., 2020)]-based brain region extractions from functional terms to evaluate overlapping brain activations and validate the correlational analysis results.

In summary, the present study had the following three hypotheses: First, game mechanics and task modifications from different games should be able to change several sub-dimensions of the gaming experiences. Second, based on correlation analysis, unique activation areas will be found in various gaming experiences. Finally, meta-analysis-based functional term decoding will demonstrate a comprehensive description of neural networks that underpin various aspects of the gaming experiences.

## Materials and methods

### Game design

Most previous studies have used existing, commercial video games, which makes it hard to manipulate specific elements of the gameplay. In addition, the games were often quite long, which can lead to potential problems with accurate *post-hoc* introspective ratings of the gaming experience. For these reasons, for the present work, I chose to develop my own set of games, which allowed me to control and manipulate various elements of the gameplay (such as the level of difficulty, stereoscopic depth, mode of input, game physics, etc.). Furthermore, all gaming sessions in the present experiment were purposefully designed to last only 5 min, which enabled participants to stay concentrated throughout the task and to provide an accurate and reliable account of their experience after playing.

**FIGURE 1:**
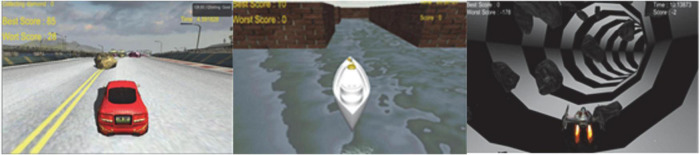
Screenshots of the experimental sessions of the car, boat, and spaceship game.

Unity3D 4.3.4f1, a 3D VR development tool^1^ was used for the present work, as well as publicly available 3D and game contents, to create three games offering different input and game dynamics (baseline assets for the games were taken from the following sources, two free and one paid one: https://www.assetstore.unity3d.com/kr/#!/content/10; http://www.youtube.com/watch?v=uWyuG4cWU_0; https://www.assetstore.unity3d.com/kr/#!/content/1869). The three games used three different vehicles (a car, a boat, and a spaceship) and required participants to complete a course while avoiding objects and/or to collect objects to be awarded bonus points (see Figure 1).

Each game had two different contexts: a training version and an experiment version. Since training on the same game may interfere with gaming experiences, the training version contained slightly different contents, but the input and game dynamics were the same across both the training and the experimental session.

During the training session, participants had to achieve a pre-set goal, which was to complete a race course with each vehicle in under 1 min (this training criterion was set after several pilot experiments were used to tune the difficulty level for the games). Importantly, since the later scanning experiment used an unusual control button layout (see below), participants were trained to control the vehicle with four adjacent keys (“asdf”) on the keyboard using their right hand.

During the experimental session, participants tried to receive a high score during the 5 min of gameplay. The goal for the car and boat games was to collect as many tokens (at 1 point per token) as possible. The tokens for the car game consisted of diamonds that were randomly placed on the racing course, while the tokens for the boat game consisted of treasure chests that were hidden in a maze that had to be navigated with the boat. In the spaceship game, the goal was to fly through a curving tunnel while avoiding obstacles (floating asteroids) as well as the tunnel wall. Each collision would result in a 1-point penalty, and the goal was to keep this score as low as possible (see Figure 1). Game scores were saved for each game session and the best and worst scores of previous players were presented on the screen to give the players additional motivation.

All games used four buttons for control, offering two degrees of freedom for moving the vehicle, and enabled stereovision through MR-compatible video goggles (NordicNeuro-Lab, NNL) controlled by Unity3D serial-port code to further enhance the players’ engagement with the games. Each game was published at a resolution of 800 × 600 pixels and at a frame rate of 60 Hz (the native resolution and frame rate of the video goggles).

### Participants

Twenty healthy, right-handed male participants who were experienced in playing video games were recruited for the functional magnetic resonance imaging (fMRI) experiment [mean age 23.5 years (SD = 2.67); all were students at Korea University]. Participants did not report any psychiatric or neurological disease or any other MRI exclusion criteria. The study was approved by the institutional ethics committee and informed consent was obtained from all participants before scanning.

### Procedure

Participants were tested for intact stereo vision by wearing Nvidia 3D glasses and watching 3D videos on a monitor to see 3D contents and then underwent a brief training session to adjust to the game controls and mechanics 1 day before the experiment. Each training session lasted 1 min, and 18 participants achieved the training goal for all games in the first session, with the remaining 2 participants needing one more training session in one of the three settings.

Before the start of the scanning experiment, participants received standard fMRI instructions. Importantly, they were instructed not to move their head during gameplay so as to avoid motion artifacts. Additional care was taken to adjust the stereo-goggles and to provide dioptric correction for each participant before scanning.

Prior to the actual experimental session, another brief training session was inserted to help participants adjust to controlling the vehicles using the MRI-compatible button box. Like in the previous training session, participants used the four fingers of their right hand (excluding the thumb). The training context was the same as on the previous day, except that gameplay was limited to 1 min. Subsequently, participants were asked whether they were comfortable with the vehicle control, which all confirmed. Finally, they went through each of the three experimental sessions. This included a 9-s resting period and 5 min of actual gameplay. Game order was counter-balanced across participants. After each game, there was a short break, after which the next session was initiated by the experimenter.

Finally, after the scanning session, participants filled out the GEQ to document their gaming experience. As described above, the GEQ contains seven dimensions (competence, immersion, flow, tension, challenge, positive affect, negative affect), with each dimension consisting of six questions that require a 1–5 (disagree-agree) Likert-type response. Participants filled out a separate questionnaire for each of the three games.

### Data acquisition

Magnetic resonance imaging data were acquired on a SIEMENS-Trio 3T scanner (Siemens Medical Systems, Erlangen, Germany) with a 12-channel SENSE head-coil (Brain Imaging Center, Korea University, Seoul, South Korea). Structural MRI images of all participants were collected using a T1-weighted, sagittal high-resolution MPRAGE-sequence [repeat time (TR) = 2250 ms, echo time (TE) = 3.65 ms, flip angle (FA) = 9°, voxel size = 1 × 1 × 1 mm^3^, 192 axial slices]. Functional imaging was performed with a gapless, echo-planar-imaging (EPI) sequence (TR = 3000 ms, TE = 30 ms, FA = 60°, voxel size = 2 × 2 × 4 mm^3^). The first 9 s of each functional run consisted of dummy scans to allow for steady-state magnetization. Since each game session lasted for exactly 5 min (excluding the dummy scans and the baseline resting period), the total number of recorded volumes for each game session was 100.

### Imaging data preprocessing

SPM12 was used to preprocesses the raw data (Wellcome Trust Centre for Neuroimaging, London, UK^2^) for the present study. First, all scans were realigned to the first volume and assessed for excessive head translations or rotations; none of the subjects exceeded the 2-mm/TR criteria. Next, the T1 image was co-registered with the mean EPI after realignment, and tissue segmentation was conducted with the SPM New Segment function. The Normalize function was then used to normalize the functional data into MNI space, to 2 × 2 × 2 mm^3^, followed by smoothing with a 6-mm FWHM Gaussian kernel.

### Behavioral data analysis

The GEQ results were first analyzed for consistency using Cronbach’s alpha and also subjected to a repeated-measure analysis of variances (ANOVAs) to test for significant differences between the seven dimensions across games. Next, inter-correlations between all different GEQ dimensions were investigated to analyze dimension dependencies; additionally, inter-correlations between the same GEQ dimensions for the different games were assessed, to analyze gaming experience similarity across games.

### Functional magnetic resonance imaging data analysis

The first analysis consisted of first-level contrasts that were run on the fMRI data by contrasting the play and the rest conditions for each participant. Based on this, a univariate, second-level analysis was run to determine active areas during gameplay. Results were thresholded at *p* < 0.05 [family-wise error (FWE)-corrected].

The second analysis investigated the relationship between the behavioral data and neural activity across the whole brain. First, a general linear model (GLM) with six head motion-related covariates was used to determine beta-estimates for each game condition. Second, pair-wise correlations between the GEQ data and the beta-estimates constructed for individual participants were assessed to construct a correlation map of the whole brain. Since the present experiment contained three different conditions, three correlation values were acquired for each of the GEQ dimensions for each voxel and each individual participant. Next, a second-level group analysis was conducted to determine whether correlation values were significantly positive or negative compared to the zero values derived from one-sample *t*-tests. A standard false discovery rate (FDR) was then applied to the resulting *p*-value maps to identify voxels that significantly correlated with each of the gaming experiences [similar to what I did in the earlier study by Ju and Wallraven (2019)].

Next, the Neurosynth decoding function was used^3^ (Yarkoni et al., 2011) to find functional correlates associated with surviving voxels for each experience. The decoding function of Neurosynth was used to conduct a meta-analysis of corresponding activation locations to extract functional correlates associated with activations which calculated articles that have a particular brain activation, articles that have a specific functional term, and probability of term from P(term| activation). At the time of writing (06/22/2022), the database contains 1,334 terms reported in 14,371 studies. To enhance my knowledge of functional terms in particular, anatomical terms were excluded and only reported functional terms associated with activations were entered into the analysis.

To complement the analysis, the NeuroQuery brain map function^4^ (Dockès et al., 2020) was used to extract activation maps from the meta-analysis of the entered functional terms. NeuroQuery uses a multivariate statistical model to predict brain regions related to the text query that first estimates the relatedness of functional terms from co-occurrence statistics and uses a linear regression model to transform term occurrences to brain activation maps. Terms related to individual gaming experiences (e.g., “game, competence”; “game, immersion”) were entered into the NeuroQuery database to extract activation maps, and the overlap between the map derived from the correlational analysis and that derived from NeuroQuery was analyzed to validate my correlation analysis results.

## Results

### Behavioral data analysis

**FIGURE 2:**
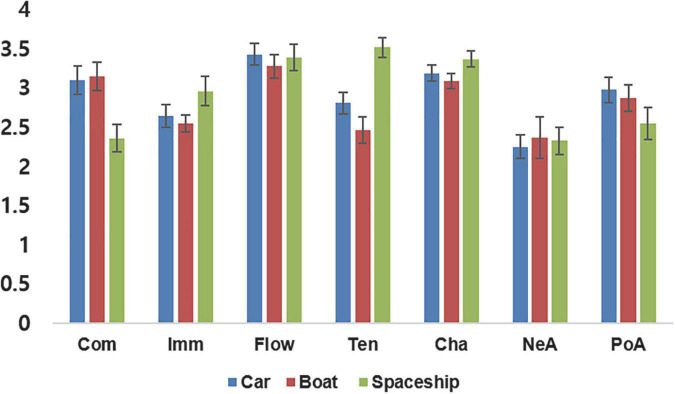
Behavioral data results. Data represent averaged gaming experiences across participants for three different games. Com, competence; Imm, immersion; Flo, flow; Ten, tension; Cha, challenge; NeA, negative affect; PoA, positive affect.

Participants’ average ratings of the three gaming experiences are summarized in Figure 2. First, Cronbach’s alpha was used to test rating consistency in GEQ responses across participants. Cronbach’s alpha for the seven gaming experiences were α = 0.84 for competence, α = 0.42 for immersion, α = 0.87 for flow, α = 0.80 for tension, α = 0.54 for challenge, α = 0.72 for negative affect, and α = 0.81 for positive affect, indicating that immersion and challenge showed relatively less consistent subjective ratings.

Next, a one-way repeated measures ANOVA was applied to investigate the effect of game type on each gaming experience dimension. Significant effects on competence, tension, and positive affect [competence: *F*_(2,18)_ = 16.86, *p* < 0.001, η2 = 0.47; immersion: *F*_(2,18)_ = 2.51, *p* = 0.095, η2 = 0.12; flow: *F*_(2,18)_ = 0.76, *p* = 0.48, η2 = 0.04; tension: *F*_(2,18)_ = 31.61, *p* < 0.001, η2 = 0.74; challenge: *F*_(2,18)_ = 2.77, *p* = 0.051, η2 = 0.20; negative affect: *F*_(2,18)_ = 0.28, *p* = 0.754, η2 = 0.03; positive affect: *F*_(2,18)_ = 3.66, *p* = 0.035, η2 = 0.27] were found, indicating that gaming experiences significantly differed across these dimensions. Next, *post-hoc* tests were conducted to further analyze differences between game conditions, and revealed significantly lower competence and tension for the spaceship compared to the car [competence: *t*(19) = 4.46, *p* < 0.001; tension: *t*(19) = 5.22, *p* < 0.001] and boat [*t*(19) = 5.18, *p* < 0.001, *t*(19) = 7.31, *p* < 0.001] conditions; positive affect was also significantly lower for the spaceship compared to the car [*t*(19) = 2.53, *p* = 0.02] condition.

**FIGURE 3:**
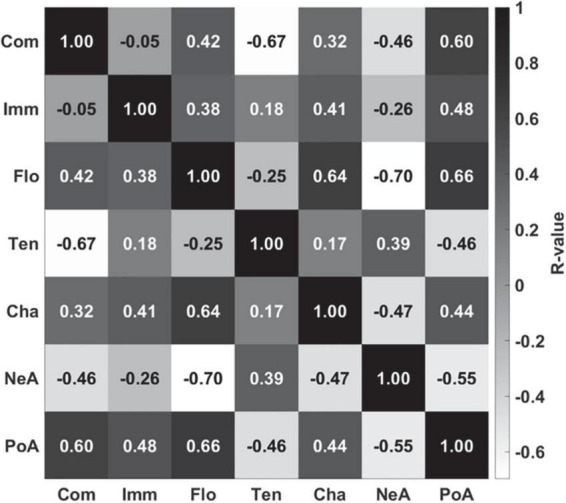
Correlations between the GEQ rating dimensions across the three different games. Com, competence; Imm, immersion; Flo, flow; Ten, tension; Cha, challenge; NeA, negative affect; PoA, positive affect.

Finally, inter-correlations between the seven GEQ dimensions were evaluated internally for correlation between same gaming experiences for different games and correlations between different gaming experiences in the same game. The highest positive correlations with positive affect were found for flow (*r* = 0.66, *p* < 0.001), competence (*r* = 0.60, *p* < 0.001), and immersion (*r* = 0.48, *p* < 0.001), indicating that the participants felt more positive affect as their feelings of competence, immersion, and flow increased. The highest correlation with challenge was found for flow (*r* = 0.64, *p* < 0.001), indicating that “getting into the game” was associated with an increase in positive feelings. Conversely, my analysis yielded the highest correlation with tension for negative affect (*r* = 0.39, *p* = 0.002), indicating that a more negative evaluation of the gaming experience went along with an increase in felt tension. As for negative correlations, competence and tension (*r* = −0.67, *p* < 0.001) as well as flow and negative affect (*r* = −0.70, *p* < 0.001) were significantly negatively correlated with each other, while the remaining three experiences, immersion (*r* = −0.26, *p* = 0.045), challenge (*r* = −0.47, *p* < 0.001), and positive affect (*r* = −0.55, *p* < 0.001) showed significant negative correlations with negative affect, indicating that an increase in negative feelings decreases the subjective experiences of immersion, flow, and challenge as well as positive affect, and that a decrease in the feeling of competence is accompanied by an increase in tension (see Figure 3 for overall correlation results and Supplementary Figure 1 for correlations between the three different game types).

### Univariate analysis

**FIGURE 4:**
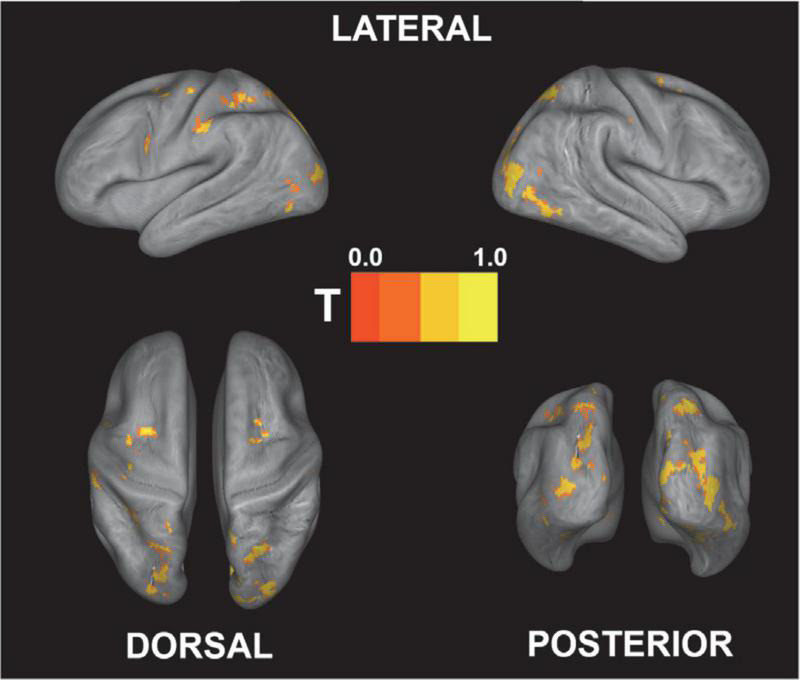
Univariate contrast for active (gameplay) vs. rest [*p* < 0.05 (FWE-corrected); the exact coordinates are presented in Table 1]. As expected, the main activation foci are located in visual and somatosensory/motor cortex regions. *T*-value is rescaled to 0–1 for better comparison.

Figure 4 shows the brain regions that were engaged more during gameplay than during rest based on the second-level univariate analysis. Not surprisingly, the activation is mainly focused in the visual cortex, as well as in the motor and somatosensory cortices. Since participants controlled the vehicles with their right hand, the analysis shows motor activations mainly centered on the left side of the brain. Additionally, the bilateral cuneus region that is involved in basic visual processing was activated (Vanni et al., 2001). The full list of significant clusters (containing more than 100 voxels) is presented in Table 1.

**TABLE 1:** Peak coordinates (in MNI space) and brain regions found to be significantly activated during active gameplay compared to rest.

X	Y	Z	Z	k (voxels)	Peak location and region (AAL)	
−4	−80	2	6.81	4299	L	Cuneus
8	−88	8	6.65		R	Cuneus
6	−84	0	6.64		R	Lingual gyrus
−38	−44	54	6.21	485	L	Inferior parietal lobule
−20	−78	30	6.15		L	Cuneus
−22	−72	42	6.10		L	Precuneus
16	−66	64	6.05	224	R	Superior parietal lobule
12	−56	60	5.74		R	Precuneus
2	−58	−24	6.71	142	R	Culmen
−4	−64	−32	5.53		L	Nodule
−26	−4	48	7.12	100	L	Middle frontal gyrus

Only clusters larger than 100 voxels are listed.

### fMRI correlational analysis

Since neural correlates of subjective gaming experiences capture rather high-level dimensions, it may be difficult to find common activations for individual game based correlational analysis. A correlational analysis between gaming experiences and neural activity across the three different game types was thus conducted, and one-sample *t*-tests with *p* < 0.05 (FDR-corrected) were used to extract the associated brain regions.

**FIGURE 5:**
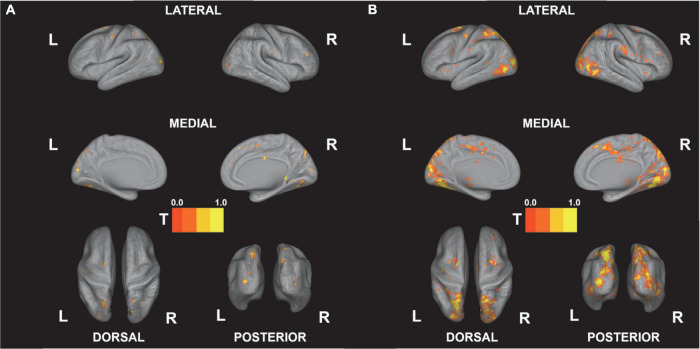
Correlations between beta estimates and behavioral gaming experiences. Results are FDR-corrected at *p* < 0.05. **(A)** Competence (negative correlation). **(B)** Tension (positive correlation). All *t*-values are re-scaled to 0–1.

Figure 5 shows the results of the correlational analysis, that is, the negative correlations for competence and the positive correlations for tension that survived after the FDR corrections (see Figure 5, *p* < 0.05 FDR-corrected). The results of the analysis show that visual, higher visual, and parietal regions including the middle occipital gyrus, lingual gyrus, cuneus, and precuneus significantly correlated with competence, while a wide range of frontal, visual, temporal, and medial regions correlated with subjective tension (see Table 2).

**TABLE 2:** Location information derived from the group-level correlational analysis.

Experiences/Relationship	Region (AAL)	Peak voxel	Z-score	Number of voxels
Competence/Negative	Cuneus	0 −90 12	4.67	169
	Lingual gyrus	10 −72 −10	4.61	305
	Middle occipital gyrus	−30 −80 6	4.55	207
	Precuneus	−18 −60 38	5.28	362
Tension/Positive	Cingulate gyrus	−16 4 50	6.92	675
	Culmen	12 −70 −14	5.58	898
	Cuneus	−26 −84 8	5.67	2,362
	Declive	−22 −54 −20	5.20	783
	Inferior frontal gyrus	48 10 32	4.30	254
	Inferior parietal lobule	−36 −42 52	4.38	257
	Inferior temporal gyrus	−48 −72 −2	5.43	137
	Insula	40 16 0	4.08	100
	Lingual gyrus	−28 −62 −8	6.79	1,799
	Medial frontal gyrus	−16 4 52	5.88	383
	Middle frontal gyrus	24 −2 48	4.96	375
	Middle occipital gyrus	−30 −82 6	7.03	1,592
	Middle temporal gyrus	50 −64 0	5.02	607
	Paracentral lobule	−8 −26 46	4.79	129
	Parahippocampal gyrus	−28 −60 −8	5.66	330
	Postcentral gyrus	−16 −56 66	4.85	440
	Posterior cingulate	22 −60 8	4.05	235
	Precentral gyrus	38 −6 44	4.62	257
	Precuneus	−20 −62 50	7.30	2,302
	Superior frontal gyrus	−22 −4 68	5.25	146
	Superior parietal lobule	−22 −60 60	6.54	636
	Thalamus	−22 −28 0	4.00	249

All analyses are FDR-corrected at *p* < 0.05 (reported clusters have more than 100 voxels). See Figure 5 for the visualization of the results.

### Meta-analysis-based decoding of functional correlates of gaming experiences

**FIGURE 6:**
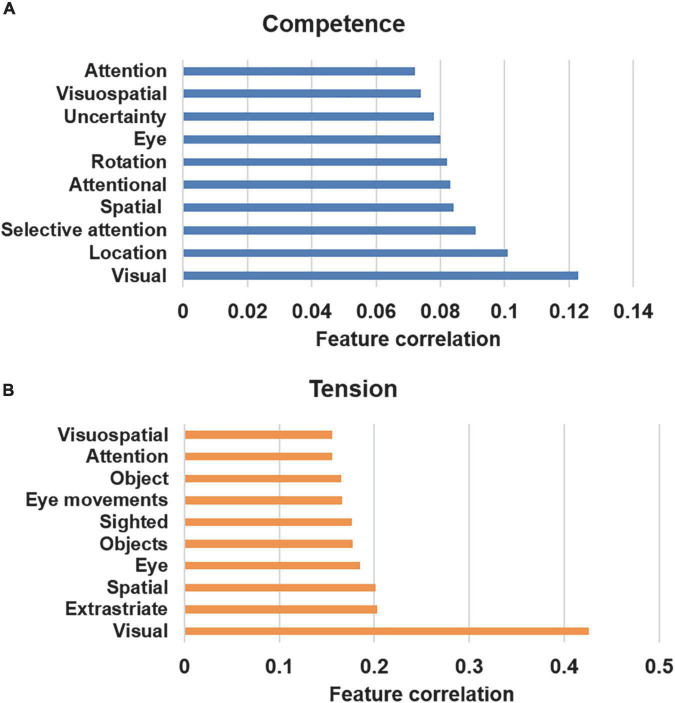
Associated psychological terms using the decoding function in Neurosynth (Yarkoni et al., 2011). **(A)** Competence (negative correlation). **(B)** Tension (positive correlation).

As the correlational analysis showed complex, whole-brain associations for subjective gaming experiences, a meta-analysis database was next used to decode feature correlations for the significant correlations that were observed for competence and tension (see Figure 6). The results of the database analysis show common functional correlates for competence and tension, including visual-, spatial-, and attention-related features. In the behavioral data, the highest negative correlations were found between competence and tension, which explains the similar decoding results for competence and tension.

**FIGURE 7:**
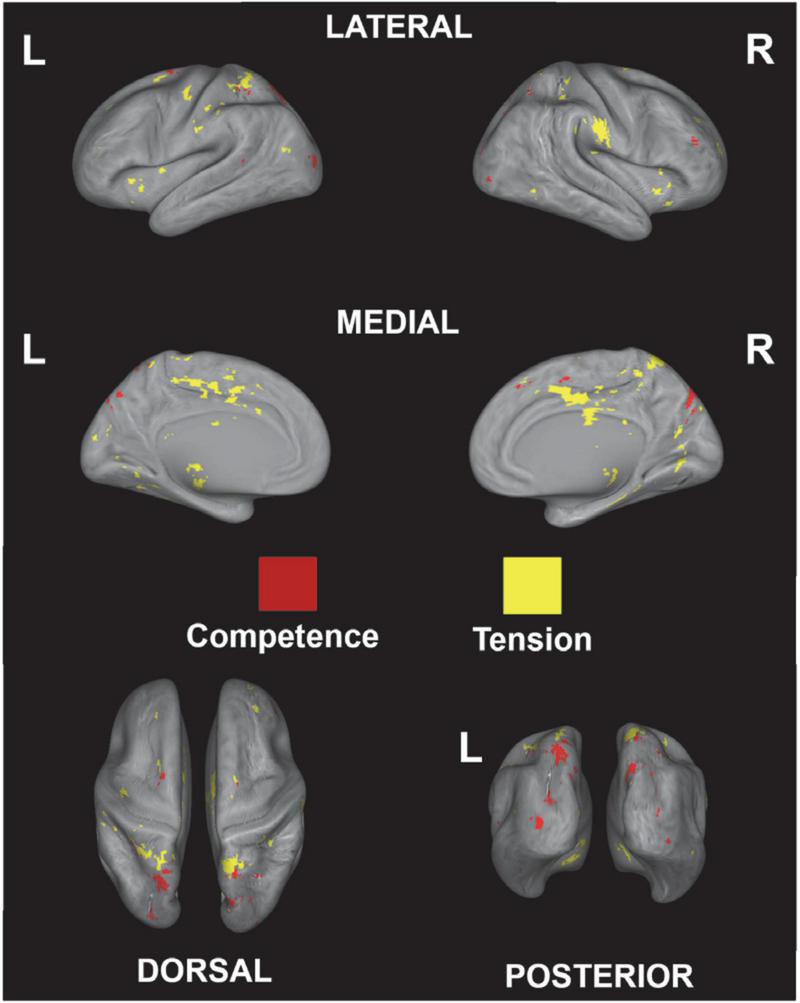
Overlapping regions derived from the correlational analysis and predicted distributions of activations from the meta-analysis using the decoding function in NeuroQuery (Dockès et al., 2020) for game competence and game tension.

Next, to validate the activation map derived from the correlational analysis, a functional term-based meta-analysis was used to investigate common activation between the correlational analysis and the meta-analysis map. The functional terms “game, competence” and “game, tension” were used to extract brain activation related to game competence and tension; overlapping regions are shown in Figure 7. The results show common activation related to competence in parietal and higher visual areas and common activation for tension in a broad range of medial regions or the whole brain (see Table 3). Overall, the term-based database meta-analysis shows an overlap in activation with the correlational analysis, which confirms the results of the latter.

**TABLE 3:** Location information derived from the overlap between the meta-analysis activation map and the correlational analysis results (reported clusters have more than 30 voxels).

Experiences	Region (AAL)	Number of voxels
Competence/Negative	Cuneus	51
	Middle occipital gyrus	102
	Precuneus	362
	Superior parietal lobule	93
Tension/Positive	Cingulate gyrus	439
	Culmen	308
	Cuneus	63
	Inferior parietal lobule	110
	Insula	90
	Lingual gyrus	65
	Medial frontal gyrus	138
	Paracentral lobule	90
	Postcentral gyrus	302
	Precentral gyrus	81
	Precuneus	127
	Superior parietal lobule	66
	Thalamus	242

See Figure 7 for the visualization of the results.

## Discussion

The present study used three different vehicle games to find brain regions associated with gaming experiences, and identified visual and motor-related region activations in a univariate analysis and visual and attention processing network associations for decoding competence and tension in a correlational analysis. Finally, a meta-analysis-based database analysis validated the correlational analysis results that revealed common activation in higher visual regions for competence and in medial and insula-related regions for tension.

### Behavioral differences across different games

In the first part of the analysis, I investigated differences in gaming experiences across different games and found that only competence, tension, and positive affect showed significant differences. A potential reason for these differences is the different control mechanism of the spaceship condition. In contrast to the car and boat games, moving the spaceship requires vertical direction control, which most participants have no experience with; this unfamiliarity with the control mechanism may have increased the difficulty level and decreased the players’ competence in the spaceship condition. In addition, trying to implement six degrees of freedom with four buttons and making sure the spaceship consistently moves forward while avoiding obstacles without any time to rest may increase subjective tension. In contrast, the boat moved relatively slower than the other two vehicles and participants might have been less concerned about time during this game. Since the definition of tension is a state of stress that makes it difficult to relax, the spaceship condition may have increased tension while the boat condition may have decreased tension, resulting in the significant difference. As a result, the lower performance and higher tension potentially decreased the players’ positive affect during the spaceship condition and led to differences in gaming experiences across conditions. However, immersion, flow, challenge, and negative affect showed no significant differences across conditions. This might be due to differences in skills between participants, which may have induced higher variability in experiences across participants, implying that the differences in experience between players were greater than the differences between games.

### Relationship between neural activity and gaming experiences

In the second part of my analysis, I used a correlational analysis to identify brain regions associated with gaming experiences. I found that competence and tension were significantly associated with neural activity, whereas no widespread correlations were found for immersion, flow, challenge, negative, and positive affect. These results are in line with the behavioral data that shows significant differences in competence and tension across conditions, and imply that to decode brain regions associated with various subjective gaming experiences, games have to provide players with significantly different experiences.

First, competence showed significant correlations with visual and higher visual areas as well as parietal regions. Several previous studies have shown associations between competence and visual areas. For instance, when adults received positive feedback about performance, in comparison to the condition of receiving negative feedback, lingual gyrus activation increased (Drueke et al., 2015). In older adults, cognitive performance about flanker tests positively correlated with left lingual gyrus. Additionally, precuneus was shown to correlate with overall competence traits (Ma et al., 2016), reduced reaction time performance (Oishi et al., 2005), and dual task performance of Parkinson’s disease patients (Wu and Hallett, 2008); moreover, the parietal cortex was found to be associated with mental calculation competence (Grabner et al., 2007) and mathematical performance (Hoppe et al., 2012), which supports the current findings that higher visual areas and the parietal cortex play an important role in the decoding of competence in the brain.

Second, visual, frontal, and medial cortex-related networks significantly correlated with subjective tension. Since tension refers to anxiety states, anxiety-related brain networks could be associated with tension-related neural activity. Previous studies have shown large gray matter volumes in the left precuneus and the right middle occipital gyrus in patients with anxiety disorder compared to controls as well as large gray matter volumes in the angular gyrus and left precuneus in patients compared to controls (Wang et al., 2018). Moreover, deficits in precuneus-related networks have been associated with social anxiety disorder (Yuan et al., 2018), which indicates a vision-related network association for subjective tension. Additionally, the anterior cingulate gyrus, the medial frontal cortex (Kimbrell et al., 1999), and connectivity between the insula and amygdala have been shown to be associated with anxiety disorder (Tian et al., 2016), suggesting that the insula and emotional processing networks contribute to the encoding of tension. Finally, a previous study showed that the inferior frontal gyrus including orbitofrontal regions is associated with tension (Lehne et al., 2013), which implies that the frontal regions also contribute to the encoding of tension. Overall, previous studies have shown that visual, frontal, and medial regions are associated with encoding tension.

However, the present study was not able to find broad significant activation foci for other gaming experiences. A potential reason for the probable lack of clear activations is the relatively small number of game types used in the present work. Since the present study targeted seven different gaming experiences, it was difficult to create distinguishable gaming experiences with only three different games. In our previous study (Ju and Wallraven, 2019), direct in-game manipulations were implemented across four different conditions, and significant results were found for four out of the seven experiences. This suggests that an increase in the number of conditions may increase the likelihood of decoding additional gaming experiences. Furthermore, our previous study used in-game context manipulations instead of changes in the games themselves and identified four different gaming experiences that were not found in the present study. This suggests that some gaming experiences can be assessed by internal manipulation and others are more likely to be identified by external manipulations. Future studies should implement both within- and between-context manipulations to decode the entire gaming experience within one study.

### Meta-analysis-based validation

Next, I used the decoding function of a meta-analysis database to identify functional correlates of competence and tension, and found the highest functional correlations for visual, attentional, and spatial processing-related terms. As attentional and visual processing were important to maintain concentration during the task and achieve a reasonable performance (Cohen and Maunsell, 2009), the identified functional correlates show that visual and attentional processing can influence subjective competence and tension.

Second, to validate the results of the correlational analysis, “game, competence” and “game, tension” were entered into a functional term-based meta-analysis to find common activation patterns. An overlap in activations of around 44.0% was found for competence and one of around 18.5% for tension. These results, showing common activations for the regions identified in the correlational analysis in the term-based meta-analysis, validate the results of the former. Specifically, the overlapping regions for competence were higher visual (Ma et al., 2016) and parietal areas (Grabner et al., 2007), which are associated with performance implementation, while those for subjective tension were frontal and medial regions including the insula, which is associated with anxiety variability (Tian et al., 2016) and auditory tension (Hilbert et al., 2014). However, compared to subjective competence, tension showed a relatively small overlap between the meta-analysis and the correlational analysis, maybe due to the fact that the term “tension” is usually associated with other sensory input, such as sound, rather than with visual stimuli, in the decoding of subjective tension (Costa, 2020). Visual task-induced tension therefore yields fewer activations in the meta-analysis database. When I changed the term from “game tension” to “visual tension” and extracted the related brain activations, the extracted map overlapped with the correlational analysis activation map to a much higher degree.

### Limitations of the study

Keeping in mind the need of enhanced understanding of neural correlates of gaming experience, the present study has several limitations that warrant future work. First, in the present study, whole brain analysis was used without a specific ROI, and therefore, results were highly exploratory and have limited affect due to lack of standardized analysis and small sample populations. However, there were only few studies (Kätsyri et al., 2013; Ju and Wallraven, 2019) that used GEQ to find associated brain regions and none of the studies found brain regions for all gaming experiences, therefore, I expect that the present study can provide a baseline to find associated brain regions from the full range of gaming experiences. Second issue of the present study is the relatively low Cronbach’s alpha value in GEQ dimension of immersion and challenge. Although the present study only found significant correlations for competence and tension, potential reason for no significant activation from immersion and challenge may come from the low reliability. Since the original GEQ study had an issue for inconsistent reliability (Law et al., 2018), to find brain regions for wider range of gaming experiences, future studies should use GEQ for dimension that has high reliability or other questionnaire like game engagement questionnaire (Brockmyer et al., 2009) to minimize potential problems. Third issue about the present study is that it only used vehicle control type games without a control condition, which makes it difficult to find effects of the game parameters including speed and performances. Since control conditions can be used to find influences of game parameters on gaming experiences, future study should add control condition to investigate the effects of game content variability and reveal the influence of in-game factors on subjective gaming experiences. Also, it is worthwhile to test other types of games including shooting game, puzzle game and role-playing game to investigate common activations across different game types.

## Conclusion

The present study investigated neural correlates of gaming experiences during three different vehicle games and used a whole-brain correlational analysis to investigate the neural correlates of the seven dimensions of the GEQ. I found wide-ranging activation patterns for two out of the seven dimensions. In addition, I used a meta-analysis database to find functional correlates of subjective gaming experiences to further validate the results of the correlational analysis. Since the present study reveals common brain activations for different games, these results can be used as a baseline to decode various gaming experiences during different types of games (e.g., shooting games, puzzle games, action games, and role-playing games) to further explore the positive and negative influences of subjective gaming experiences on the brain.

## Data availability statement

The raw data supporting the conclusions of this article will be made available by the authors, without undue reservation.

## Ethics statement

The studies involving human participants were reviewed and approved by Korea University. The patients/participants provided their written informed consent to participate in this study.

## Author contributions

The author confirms being the sole contributor of this work and has approved it for publication.

## Footnotes

1
http://www.unity3D.com

2
http://www.fil.ion.ucl.ac.uk/spm/

3
http://neurosynth.org/analyses/terms

4
https://neuroquery.org/
