# Supplementary material for: Identifying neural correlates of multidimensional, subjective gaming experiences during active gameplay
Source: Front Hum Neurosci. 2022 Oct 28;16:1013991. doi: 10.3389/fnhum.2022.1013991 (PMC9651959; doi:10.3389/fnhum.2022.1013991)
Supplement: Supplementary file 1 [file Data_Sheet_1.pdf]

frontiers

Supplementary Material

1 Supplementary Data

Fig. S1 shows correlations between the three different game types.

1.1 Supplementary Figures

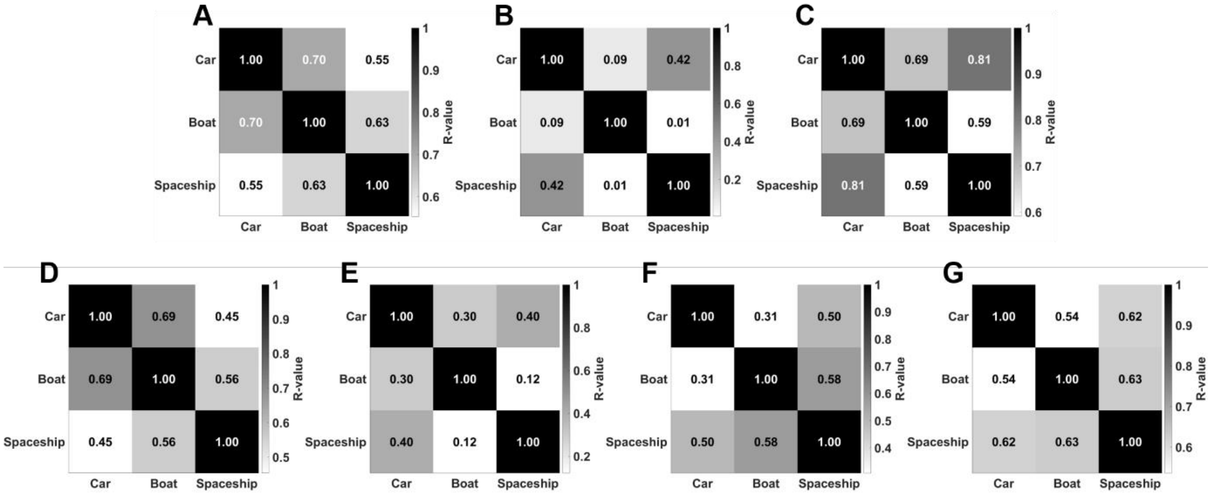

Figure S1. Correlations within the GEQ rating dimensions between the three different game types. **A:** competence, **B:** immersion, **C:** flow, **D:** tension, **E:** challenge, **F:** negative affect, **G:** positive affect.
